# Synergistic effect of umbilical cord extracellular vesicles and rhBMP-2 to enhance the regeneration of a metaphyseal femoral defect in osteoporotic rats

**DOI:** 10.1186/s13287-024-03755-8

**Published:** 2024-05-20

**Authors:** Amelie Deluca, Andrea Wagner, Patrick Heimel, Christian Deininger, Florian Wichlas, Heinz Redl, Eva Rohde, Herbert Tempfer, Mario Gimona, Andreas Traweger

**Affiliations:** 1 Institute of Tendon and Bone Regeneration, Salzburg, 5020 Austria; 2 Department of Traumatology, KABEG—Klinikum Klagenfurt am Woerthersee, Klagenfurt, 9020 Austria; 3 Austrian Cluster for Tissue Regeneration, Vienna, 1200 Austria; 4 Ludwig Boltzmann Institute for Traumatology, The Research Centre in Cooperation with AUVA, Vienna, 1200 Austria; 5 Department of Orthopedics and Traumatology, Salzburg University Hospital, Paracelsus Medical University, Salzburg, 5020 Austria; 6 GMP Unit, Spinal Cord Injury and Tissue Regeneration Centre Salzburg, Paracelsus Medical University, Salzburg, Austria; 7 Department of Transfusion Medicine, Salzburger Landeskliniken GesmbH, Paracelsus Medical University, Salzburg, Austria; 8 Research Program “Nanovesicular Therapies”, Paracelsus Medical University, Salzburg, Austria; 9 Core Facility Hard Tissue and Biomaterial Research, Karl Donath Laboratory, University Clinic of Dentistry, Medical University Vienna, Vienna, Austria

**Keywords:** BMP-2, Extracellular vesicles, Exosomes, Drug delivery; osteoporosis, Metaphyseal defect

## Abstract

**Background:**

The aim of this study was to evaluate potential synergistic effects of a single, local application of human umbilical cord MSC-derived sEVs in combination with a low dose of recombinant human rhBMP-2 to promote the regeneration of a metaphyseal femoral defect in an osteoporotic rat model.

**Methods:**

6 weeks after induction of osteoporosis by bilateral ventral ovariectomy and administration of a special diet, a total of 64 rats underwent a distal femoral metaphyseal osteotomy using a manual Gigli wire saw. Defects were stabilized with an adapted Y-shaped mini-locking plate and were subsequently treated with alginate only, or alginate loaded with hUC-MSC-sEVs (2 × 10^9^), rhBMP-2 (1.5 µg), or a combination of sEVs and rhBMP-2 (*n* = 16 for each group). 6 weeks post-surgery, femora were evaluated by µCT, descriptive histology, and biomechanical testing.

**Results:**

Native radiographs and µCT analysis confirmed superior bony union with callus formation after treatment with hUC-MSC-sEVs in combination with a low dose of rhBMP-2. This finding was further substantiated by histology, showing robust defect consolidation 6 weeks after treatment. Torsion testing of the explanted femora revealed increased stiffness after application of both, rhBMP-2 alone, or in combination with sEVs, whereas torque was only significantly increased after treatment with rhBMP-2 together with sEVs.

**Conclusion:**

The present study demonstrates that the co-application of hUC-MSC-sEVs can improve the efficacy of rhBMP-2 to promote the regeneration of osteoporotic bone defects.

**Supplementary Information:**

The online version contains supplementary material available at 10.1186/s13287-024-03755-8.

## Introduction

The prevalence of osteoporosis (OP), especially in postmenopausal women is steadily increasing and already affects more than 20 million individuals in the USA alone [1]. It is characterized by low bone mineral density (BMD), the deterioration of bone architecture which results in reduced bone strength and hence, increased susceptibility to fractures [2, 3]. The poor healing of such fractures is associated with a significantly increased patient morbidity and mortality [4].

Generally, failure of fractures to heal adequately leads to complications that are especially present in the elderly, such as pain, weakness, reduced mobility and deterioration of the overall health status [5]. Therefore, the development of novel effective therapies to improve osteoporotic fracture repair is in dire need. Next to bone grafting and highly sophisticated biomimetic scaffolds, platelet-rich plasma (PRP), various growth factors, and multipotent mesenchymal stromal cells (MSCs) have been utilized in preclinical and clinical studies to enhance bone repair [6, 7]. Although the application of MSCs has clearly been shown to improve bone repair [8], the benefits of autologous cell therapies are significantly reduced in the elderly [9], the largest target group for osteoporotic fracture treatment.

More recently, MSC-derived trophic factors, including secreted vesicles, are considered to mainly contribute to the efficacy of cell therapies. Small extracellular vesicles (sEVs) are nano-sized lipid-bound vesicles released from cells into the extracellular space [10]. Initial evidence that sEVs exert a therapeutic effect was provided by Bruno S. et al., who demonstrated that human bone marrow-derived MSC-sEVs were as effective as their parental cells in promoting kidney regeneration in a murine acute kidney injury model [11]. Since then, a number of preclinical studies have demonstrated the potential of MSC-derived sEVs to promote musculoskeletal tissue repair [12], making them a promising contender for the treatment of bone disorders [13]. Interestingly, the co-administration of sEVs has also been demonstrated to amplify the efficacy of anti-cancer drugs in vitro [12] and to effectively reduce experimentally induced intrauterine adhesions in vivo when delivered together with estrogen [14]. Therefore, sEVs might also have the potential to enhance the efficacy of established bone repair therapeutics, including the administration of growth factors such as recombinant BMP-2.

Bone morphogenetic protein-2 (BMP-2), a member of the TGF-β superfamily, is a potent osteoinductive cytokine and promotes MSC proliferation and differentiation [15]. Several clinical studies have been conducted to assess the safety and efficacy of recombinant BMP-containing devices for treatment of diaphyseal bone fractures, delayed union, tibial nonunion and spinal fusion [16]. However, as supraphysiological doses have mostly been applied, local and systemic adverse events were reported, yielding an undesired clinical outcome. Therefore, strategies to lower the required dose of BMP-2 without a concurrent loss in efficacy are under investigation and of great interest.

The combination of osteoinductive scaffolds with umbilical cord-derived mesenchymal stem cell-secreted extracellular vesicles (UC-MSC-sEVs) has previously shown promising results in enhancing in vivo bone repair [17, 18]. While this approach has demonstrated efficacy, there is an intriguing potential for the combination of human hUC-MSC-sEVs with a low dose of recombinant human (rh)BMP-2 to further enhance bone healing. This strategy not only could improve the therapeutic outcome, but also has potential to mitigate any safety concerns associated with the use of higher doses of rhBMP-2. Hence, the primary objective of the current investigation was to explore the possible effects of hUC-MSC-sEVs in amplifying rhBMP-2-mediated bone repair, particularly in the context of osteoporotic bone, employing a metaphyseal defect model in rat femurs.

## Materials and methods

### Experimental animals

All animal experiments and conducted procedures were in accordance with the law on animal experimentation and are approved by the regulatory authorities. The work has been reported in line with the ARRIVE guidelines 2.0.

A total of sixty-four 12-week-old, adult female Sprague Dawley rats (Janvier Labs SAS, France) weighing approximately 300–350 g were randomly assigned to either one of four experimental groups of equal size (*n* = 16; see 2.2 for details). The animals were kept under standard housing conditions (2–3 rats per cage) with free access to food and water. Post-operatively the same animals were kept in groups of 2 to 3 rats per cage. Rooms were maintained at 25 ± 2 °C and a 12:12 h light/dark cycle, light on at 07:00 h.

### Animal study design and surgical procedures

**Fig. 1:**
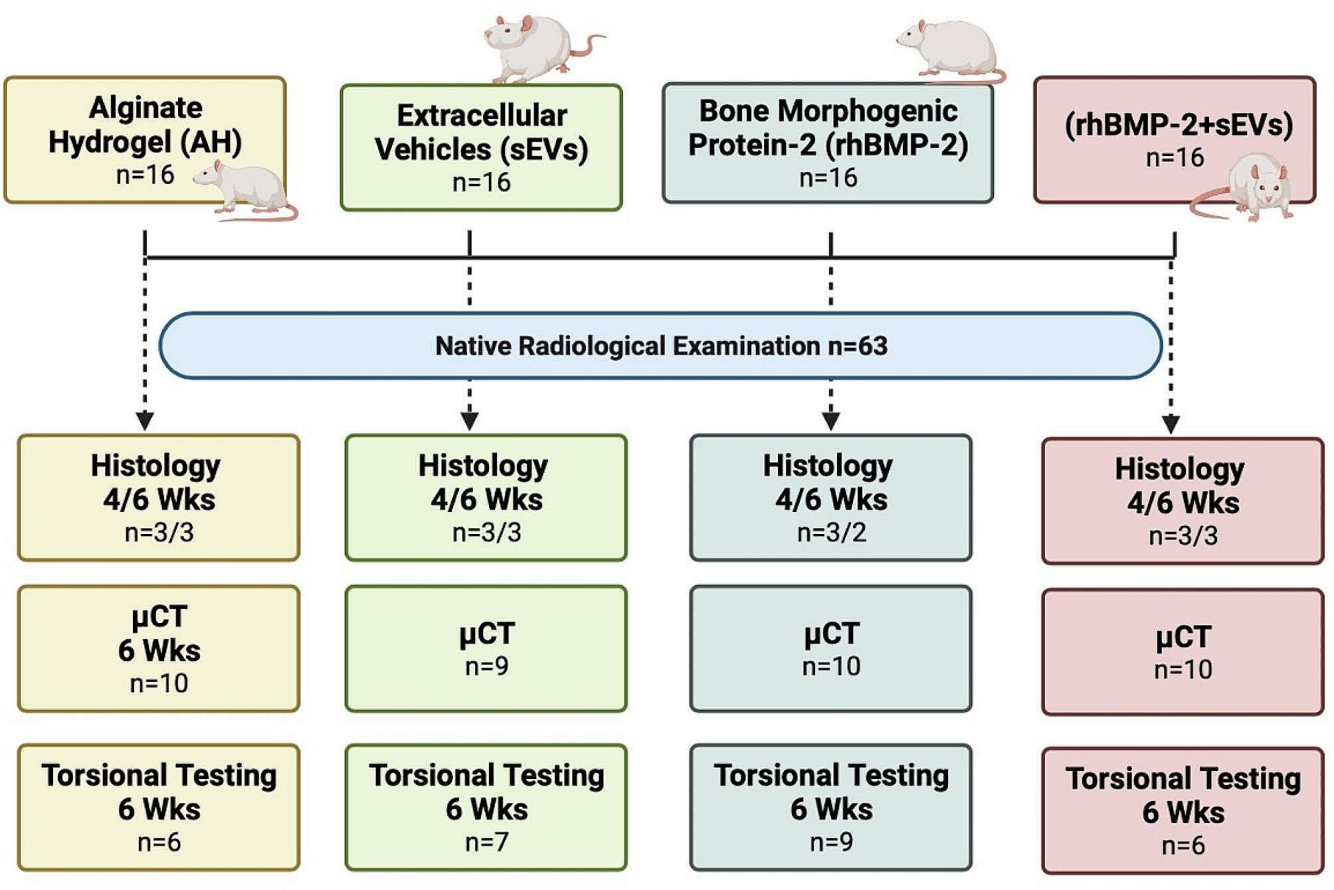
Overview of groups according to the applied biomaterials and used follow-up procedures including native radiographs, histology, micro computed tomography (µCT), analysis, and torsional testing. Wks = weeks

After an appropriate acclimatization phase, all rats underwent a ventral ovariectomy (OVX, *n* = 64) and osteoporosis was further induced by feeding a calcium-, phosphorus-, vitamin D3-, soy- and phytoestrogen-free diet (Altromin-C1034, Altromin Spezialfutter GmbH, Lage, Germany) for a total of six weeks as previously described in Deluca et al. [17]. Subsequently, all animals underwent right femoral surgery to create an osteotomy gap with a Gigli wire saw as published [17]. Briefly, thirty minutes preoperatively all rats received 0,03 mg/kg buprenorphine as a subcutaneous (s. c.) injection and inhalation anesthesia (isoflurane) was induced. Body temperature was maintained using a heating pad (Harvard Apparatus, Holliston, MA, USA) and prior to surgery, each animal received an antibiotic (Clindamycin s.c., 45 mg/kg). After aseptic preparation, the femur was exposed from the lateral femoral condyle to the lateral midshaft area. The capsule of the knee joint was opened, and the patella dislocated medially. Prior to performing the osteotomy, the femur was stabilized using an adapted Y-shaped interlocking plate (Veterinary Orthopedic Implants Inc., 1.5 mm Condylar Angle Stable, DT Locking 2 × 6 hole; Orly, France). Subsequently, a distal metaphyseal osteotomy was set with a 0.66 mm Gigli wire saw (RISystem AG, Lanquart, Switzerland). The site was thoroughly rinsed with sterile saline solution. Defects were then treated according to the following groups (see also Fig. 1): Alginate hydrogel only (AH), AH + hUC-MSC-sEVs (sEVs; dose: 2 × 10^9^), AH + rhBMP-2 (dose 1.5 µg; Peprotech, Vienna, Austria), AH + rhBMP-2 + sEVs (2 × 10^9^ and 1.5 µg respectively). The final volume of the implanted alginate clot was 80 µl using alginate at a final concentration of 1.5 mg/ml (PRONOVA SLG-20; NovaMatrix, Sandvika, Norway). Wound closure was performed in layers with closure of the joint capsule, muscle sutures and sterile surgical clips (FST, Heidelberg, Germany). Immediately post-operatively, a control X-ray was performed to confirm the accurate placement of the osteosynthesis. As postoperative analgesia the animals received a daily dose of buprenorphine (0.03 mg/kg, twice daily) for a total of 72 h and oral tramadol-hydrochloride (20 mg/kg body weight, once daily) via their drinking water for a total of 7 days. The animals had free access to food and water and were frequently monitored for any complications, weight loss or abnormal behavior.

Four- and six-weeks post-treatment rats were euthanized were euthanized by intracardial barbiturate injection (Pentobarbital-Natrium 300 mg/mL; Release, WDT, Germany) under general isoflurane anesthesia and the femurs were harvested and prepared for subsequent µCT, biomechanical, and histological analysis.

### Preparation of hUC-MSC-sEVs

Umbilical cord material was gathered following the uncomplicated delivery of healthy newborns from women who had previously provided written informed consent during an earlier stage of pregnancy (Department of Obstetrics and Gynecology; University Clinic Salzburg). The approval to use human umbilical cord MSCs was obtained from the Ethics Committee of the province of Salzburg (protocol 415-E/1776/4-2014). Following delivery, cords were promptly collected and preserved in phosphate-buffered saline (PBS) for subsequent processing as previously described [18]. The entire cords were washed in PBS to remove contaminating blood cells, after which the cord stroma was cut into 1–2 mm³ fragments. These pieces were then placed in a culture plate, allowing them to adhere before adding culture medium - α-MEM (Sigma-Aldrich) supplemented with 10% (v/v) pooled human platelet lysate (pHPL) and Dipeptiven (5.5 mg/mL, Fresenius-Kabi, Graz, Austria). After 10–12 days, visible UC-MSC colonies had formed and the remaining cord tissue fragments were removed. UC-derived MSCs were enzymatically detached using TrypLE Select CTS (A12859-01, Gibco) and subsequently expanded in cell factory systems (CF4, Thermo Scientific). Immunophenotype and viability analysis of MSCs were conducted following the marker profile suggested by the International Society of Cell Therapy (ISCT) in 2005. hUC-MSCs were expanded from cryopreserved stocks in a fibrinogen-depleted culture medium (α-MEM supplemented with 10% v/v pooled human platelet lysate and 5.5 mg/mL dipeptiven; Merck, Vienna, Austria) and subsequently, upon reaching 60–70% confluency the growth medium was exchanged with vesicle-depleted harvest medium (α-MEM supplemented with 5% v/v pooled human platelet lysate, 5.5 mg/mL dipeptiven). Cells were cultured for a total of 24 h and culture supernatants were collected, centrifuged at 2.500×g for 20 min to pellet cell debris and the supernatants were then sterile filtered (0.22 μm; Merck, Vienna, Austria). Processed culture media were concentrated by tangential flow filtration (TFF) (100 kDa hollow fiber filter, Spectrum Labs-Repligen, Breda, The Netherlands) and buffer-exchanged into sterile PBS by diafiltration. Finally, hUC-MSC-sEVs were collected by ultracentrifugation (120 000×g, 3 h at 18 °C). hUC-MSC-sEVs were resuspended in sterile Ringer’s lactate at a final concentration of 2 × 10^9^ particles per 15 µl. Particle size and amount were determined using a nanoparticle tracking (NTA) device in light scatter mode (ZetaViewPMX 110, Particle Metrix; Inning am Ammersee, Germany) and total protein content was determined by fluorescence spectroscopy (Qubit 3.0; Life Technologies; Vienna Austria). Finally, sEV surface protein profiling was conducted using the MACSPlex Exosome Kit according to the manufacturer’s instructions (Miltenyi Biotec; Bergisch Gladbach, Germany).

### Histological examination and staining

Four- and six-weeks post-treatment three animals (*n* = 3) of each group were euthanized and the right femur, including muscle tissue, was explanted by exarticulation at the knee and hip joint and were then fixed in 4% paraformaldehyde (PFA) in PBS at 4–6 °C. For the AH + rhBMP-2 group one animal died after surgery (see results section) and therefore for the time point 6 weeks after surgery only 2 samples were available. After 48 h, samples were decalcified in 2% PFA/ 12.5% EDTA solution (pH = 7.5). After a minimum of 7 weeks the femora were evaluated by X-ray to ensure complete decalcification. After removing the screws and osteosynthesis plate, samples were then processed for paraffin embedding. 6 μm sections were prepared and deparaffinized using Roti®-Histol (Carl Roth, Germany), rehydrated in a graded alcohol series and stained with Masson-Goldner trichrome stain or Alcian blue/Nuclear Fast Red Acid [19] according to standard procedures. Digital high-resolution images were acquired on an Olympus VS120 slide scanner (Olympus, Vienna, Austria).

### Radiological evaluation and µCT analysis

Healing was monitored by two independent observers. Anteroposterior and lateral radiological views were acquired under general anesthesia immediately post-operatively, after 1 and 2 weeks and then at biweekly intervals for all animals.

After fixation in 4% PFA in PBS, osteosynthesis plates were carefully removed, ensuring no fracture occurred. One sample of the AH-sEV group was excluded, as the femur broke during preparation (see Fig. 1). During µCT scanning, explanted femora were stored in polymer sample tubes filled with formalin to prevent dehydration. Of each group, nine to ten (*n* = 9–10) samples were scanned in a SCANCO µCT 50 (SCANCO Medical AG, Brüttisellen, Switzerland) at 70 kVp, 114 µA filtered with 0.5 mm Al. Over a field of view of 20.4 mm, 850 Projections/180° were integrated for 475 ms with averaging 1, HW-binning 2 and reconstructed to an isotropic resolution of 12 μm. Scans were exported as DICOM slices calibrated to mgHA/cm³. Image preprocessing and measurements were performed using Using Fiji [20] and Definiens Developer XD 2.7 (Definiens AG, Munich, Germany). Bone Volume (BV) over total Tissue Volume (TV) was determined for the defined, encompassing the newly formed bone in the medullary canal, within the osteotomy region, and the periosteal callus within a defined ROI (for further details see supplementary information). BV/TV are expressed in %.

### Radiological and histological scoring

All native radiographs, µCT images, and histological sections were evaluated by three independent observers (Author 1, 4, and 10) who were blinded to the groups. The radiographs and µCTs were scored for callus formation, quality of union, and bone remodeling after 6 weeks as previously described [17, 21] and summarized in Table 1. The maximum expected total score is 8 for bone fracture repair. The histological sections were evaluated by the same investigators for fracture healing and scored as summarized in Table 2 [17, 21] at 4 and 6 weeks after treatment. The maximum total histological score for bone fracture healing achievable is 12.

**Table 1:** Radiographic scoring system for fracture healing

Categories	Scores			
	3	2	1	0
Periosteal reaction	Complete across defect	Moderate (> 50%)	Mild (< 50%)	None
Bone union	Complete bony union	Moderate (> 50%)	Mild (< 50%)	None
Remodeling		Complete remodeled cortex	Mild (< 50%)	None

**Table 2:** Histological scoring system for fracture healing

Categories	Scores			
	3	2	1	0
Callus formation	Complete across defect	Moderate (> 50%)	Mild (< 50%)	None
Bone union	Complete bony union	Moderate (> 50%)	Mild (< 50%)	None
Cortex remodeling	Complete remodeled cortex	Moderate (> 50%)	Mild (< 50%)	None
Marrow changes	Adult type fatty marrow	Moderate (> 50%)	Mild (< 50%)	None

### Biomechanical analysis

After µCT was performed, samples underwent torsion testing using an adapted universal tensile testing machine (Zwick / Roell; Ulm Germany). Therefore, the femora were proximally and distally embedded in resin (RenCast® FC 53; Biesterfeld AG, Hamburg, Germany), ensuring the embedding was along the longitudinal axis of the diaphysis and without any contact to the defect area. Misaligned samples were excluded. After applying a preload of 0.2 N, samples were loaded at a constant speed of 0.1 mm/sec. The endpoint was set as a fracture of the femora which occurred in the pre-determined metaphyseal fracture zone. Samples fracturing at a different region were excluded from the analysis. Torque (Nmm) and torsional stiffness (Nmm/°) were determined from the torque vs. rotation curve. Tissue stiffness was determined from a linear region between a rotation angle of 0.5° and 1.5°.

### Statistical analysis

All obtained data samples are reported as means ± standard deviations. Datasets were tested for normal distribution using the Shapiro Wilk test. A one-way ANOVA test with post-hoc pairwise.

comparison (Tukey’s) or for pairwise comparisons an unpaired t-test was used. Statistical significance was set at *P* = 0.05. All tests were performed using GraphPad Prism v. 9.02 (La Jolla, CA, USA).

## Results

### Characterization of hUC-MSC-sEVs

**Fig. 2:**
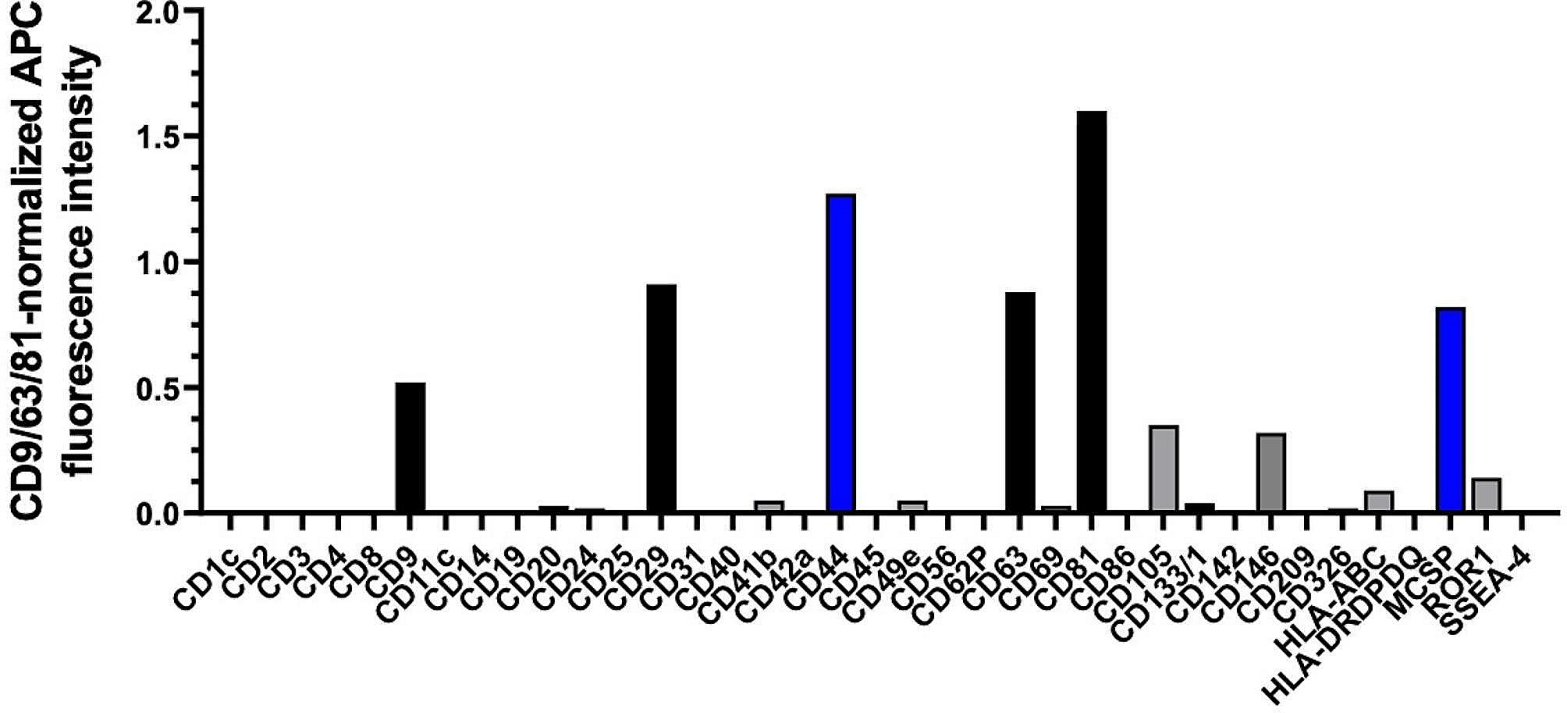
MASCSPlex profiling of hUC-MSC-sEV preparation. Bar graph shows the robust expression of the sEV marker proteins CD9, CD29, CD63, and CD81 (black bars). In addition, the MSC-EV-specific markers CD44 and MSCP/NG2 were clearly present (blue bars)

Nanoparticle tracking analysis (NTA) revealed a mean sEV size of 139 nm and total protein content was 1050 µg/mL. Bead-based multiplex analysis revealed the robust expression of the sEV tetraspanins CD9, CD63, and CD81 in addition to CD29 (Integrin beta- 1), the MSC-EV-specific markers CD44 (hyaluronic acid receptor) and MSCP/NG2 (melanoma‐ associated chondroitin sulfate proteoglycan) (see Fig. 2).

### General Animal health

In total, 63 out of 64 animals reached the endpoint of 6 weeks after the second surgery. One animal (rhBMP-2) died shortly after the metaphyseal defect surgery for unknown reasons. Overall, no severe intra-operative complications occurred and all animals showed normal behavior, with full weight bearing 24 h after surgery. No animals were excluded in the further course due to weight loss or other signs of postoperative complications.

### Radiographic evaluation

**Fig. 3:**
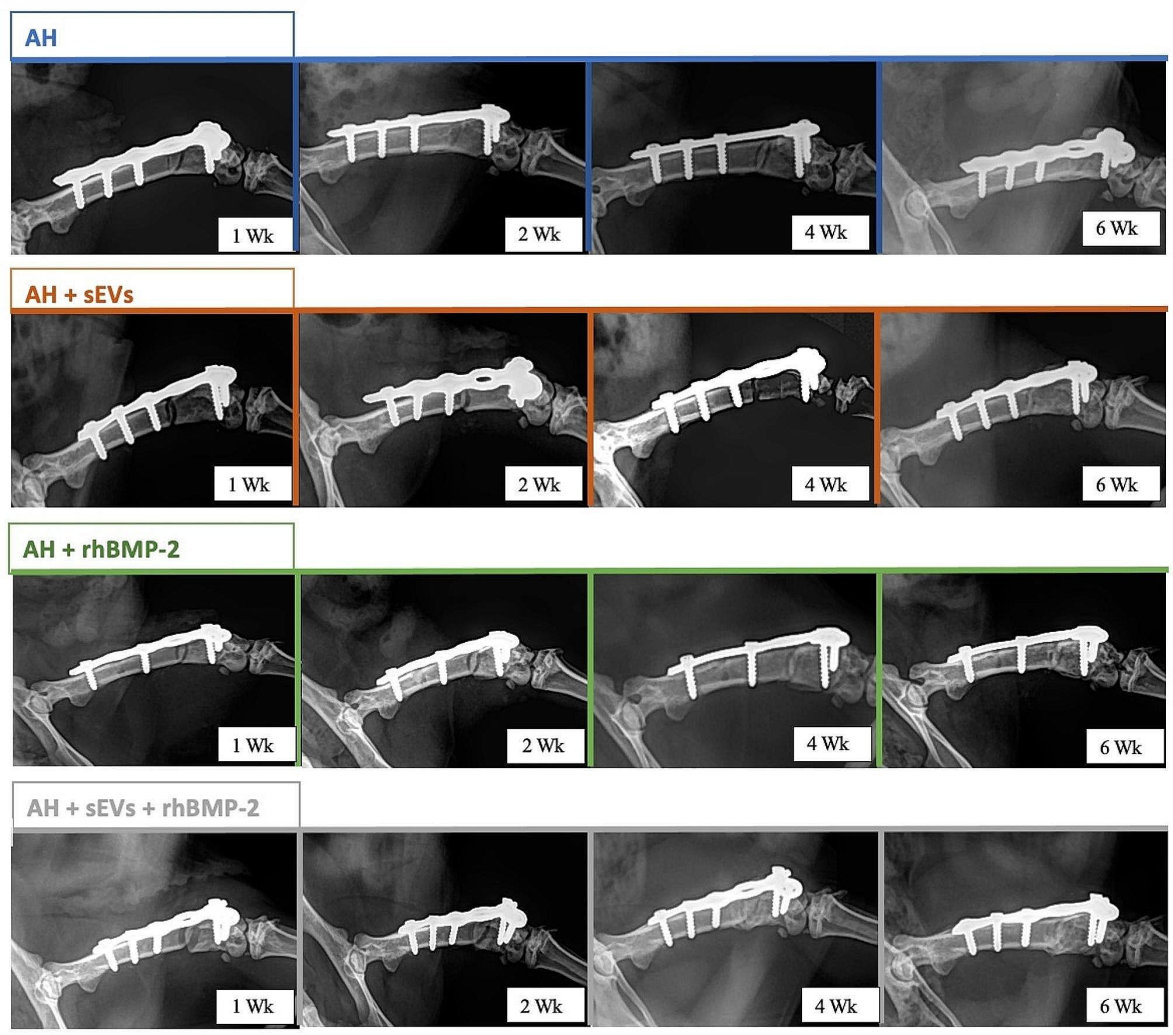
: Radiological follow-up 1-, 2-, 4- and 6-weeks post-surgery after distal femoral plate fixation. The defect was treated with either alginate only (AH), alginate with huUC-MSC-sEVs (AH + sEVs), alginate with rhBMP-2 (AH + rhBMP-2) or alginate with a combination of BMP-2 + sEVs (AH + rhBMP-2 + sEVs)

The obtained X-rays immediately post-surgery, after 1 week, and followed by biweekly intervals were analyzed and documented for comparison (Fig. 3). Defects created with the 0.66 mm Gigli saw and treated with AH only demonstrated minimal bony bridging with minimal to no callus formation. Defects treated with AH + sEVs showed moderate bony bridging with minimal callus formation. Similarly, the application of AH with a low dose of rhBMP-2 also yielded bony bridging identical to the bridging observed in the femora treated with AH + sEVs. A persistent non-union was not observed for any of the samples. However, significant callus and bone formation was mainly observed 6 weeks after addition of alginate hydrogel loaded with a combination of a low dose of rhBMP-2 and hUC-MSC-derived (Fig. 3, bottom row).

### Histology

**Fig. 4:**
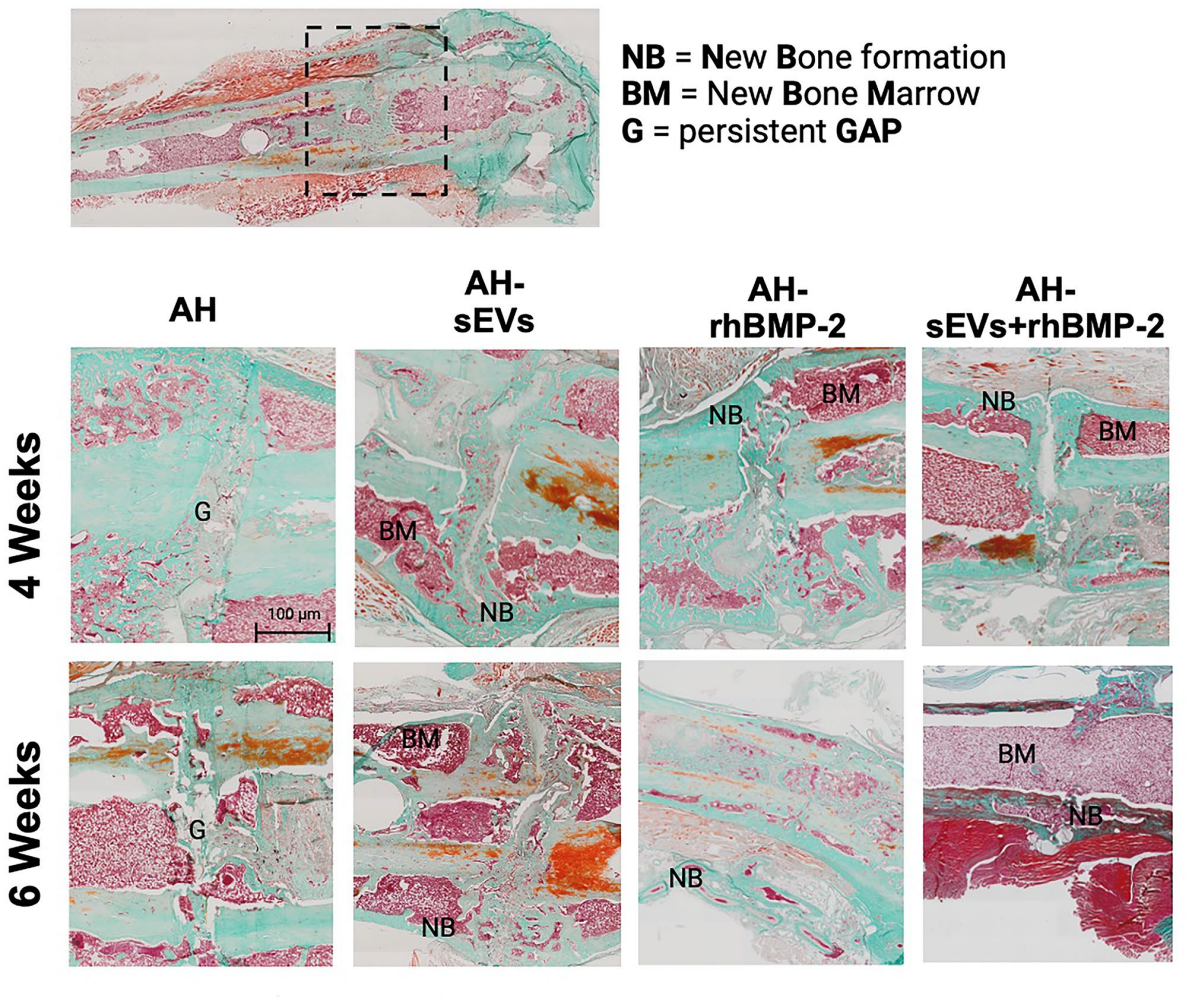
: Histological evaluation 4 and 6 weeks after treatment of Masson Goldner trichrome-stained FFPE sections. Dashed lines indicate the defect area. Scale bar = 100 μm

4 weeks after treatment of the defects, for all samples only minor cortical bridging and callus formation were evident by descriptive histology. 6 weeks post-treatment, femora treated with AH samples presented upon histological staining with partially extensive callus formation but little bony bridging. In contrast, the bony bridge after application of AH + sEVs was more enhanced with bony callus formation and active bone remodeling within the defect area. For AH + rhBMP-2-treated femora an increasing osseous continuity and less callus formation was seen in Masson Goldner Trichrome-stained (Fig. 4) and by Alcian blue-stained (see Suppl. Figure 1) FFPE sections. Sections from AH-rhBMP-2 + sEVs-treated samples displayed complete remodeled cortices and minimal callus formation. Together, the histological examination was concurrent with the radiographic findings.

### Microcomputed tomography

**Fig. 5:**
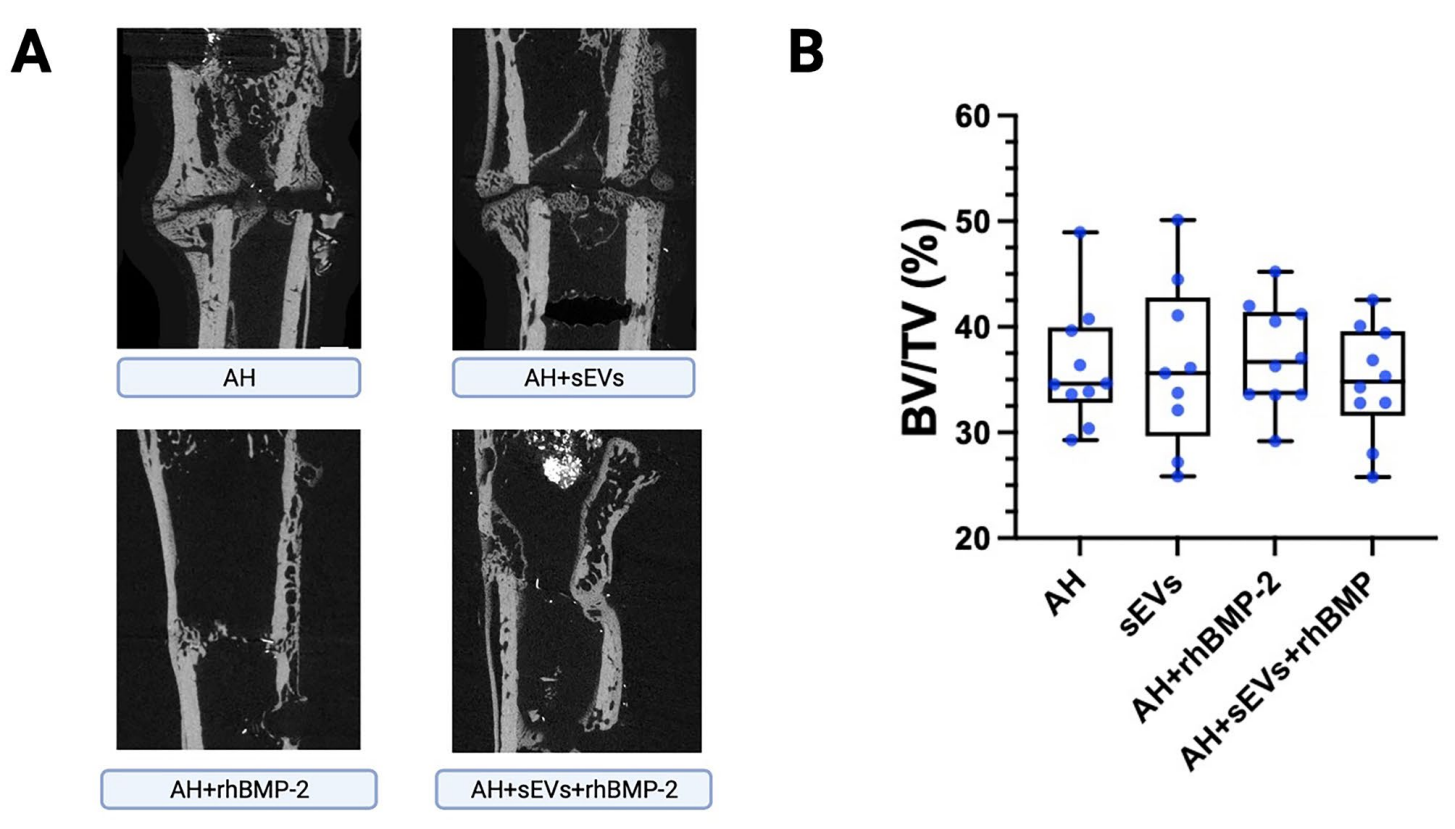
µCT images of the defect area 6 weeks after treatment. (**A**) Representative sagittal µCT sections of the defect area. (**B**) Quantitative analysis of BV/TV for all 4 treatment groups, revealing no significant difference between the newly formed bone volume

BV/TV was determined for the defect area, including the callus volume and the newly formed bony tissue within the medullary canal (see Suppl. Figure 2 for segmentation strategy). However, there were no significant differences evident when comparing the volumes of the newly formed bone tissue (Fig. 5A/B). Analysis of sagittal µCT sections however confirmed the overall findings by descriptive histology and radiography. None of the samples of the AH group showed a full consolidation of the defect, only a mild periosteal reaction with initial bony remodeling and incipient callus formation. Samples in the AH + sEVs group showed fracture healing by mild to moderate callus formation. The fracture itself however exhibited no bony union with the cortices being largely open. Animals treated with rhBMP-2 also showed mild to moderate callus formation and only one out of ten samples revealed complete bony bridging. Analyzed sections from samples treated with rhBMP-2 in combination with hUC-MSC-sEVs presented with more extensive callus formation, a moderate to complete periosteal reaction around the defect and moderate to complete bony union. Representative images are shown in Fig. 5A.

### Radiological and histological scoring

To further determine the extent of bony union, all histological Sect. (6 weeks post surgery) and radiographic images (native radiographs and µCT sections combined) were evaluated for bone repair as described in the methods section, further substantiating our findings by qualitative analysis (summarized in Table 3). Histological scoring for the AH group showed partially extensive callus formation, no union with mild cortex remodeling, and a mean total histological score of 5 ± 1. Samples treated with AH-sEVs presented with moderate to extensive callus formation, moderate bony union, extensive remodeling of the cortices and the marrow with a mean score of 7 ± 1. Femora after application of AH-rhBMP-2 to the defect showed widespread remodeling with callus formation, pronounced bony union at the fracture site (full union evident for 1 out if 10 samples) with cortex remodeling and abundant marrow changes with new bone formation; mean total score 9 ± 1. Defects treated with AH-rhBMP-2 + sEVs demonstrated mostly complete bony union, marrow remodeling to the consistency of regular healthy bone tissue, moderate callus formation with a mean total histological score of 11 ± 1 (summarized in Table 3).

Analyzing the radiological mean scores of samples in the AH group, resulted in a mean total score of 5 ± 1 with a complete periosteal reaction and mild bony union. Samples of the AH-sEVs and AH-rhBMP-2 group were classified with a radiological mean total score of 6 ± 1. The analysis of the native radiographs demonstrated a complete periosteal reaction, moderate bony union and incipient remodeling with callus formation. Femurs treated with AH-rhBMP-2 + sEVs yielded a mean total score of 8 ± 1, with a complete periosteal reaction and new bone formation, broad fracture union, and remodeling with additional callus formation around the fracture site.

**Table 3:** Summary of obtained histological and radiological mean scores

	AH	AH-sEVs	AH rhBMP-2	AH-rhBMP-2 + sEVs
Histological mean score	5 ± 1	7 ± 1	9 ± 1	11 ± 1
Radiological mean score	5 ± 1	6 ± 1	6 ± 1	8 ± 1

### Biomechanical analysis

**Fig. 6:**
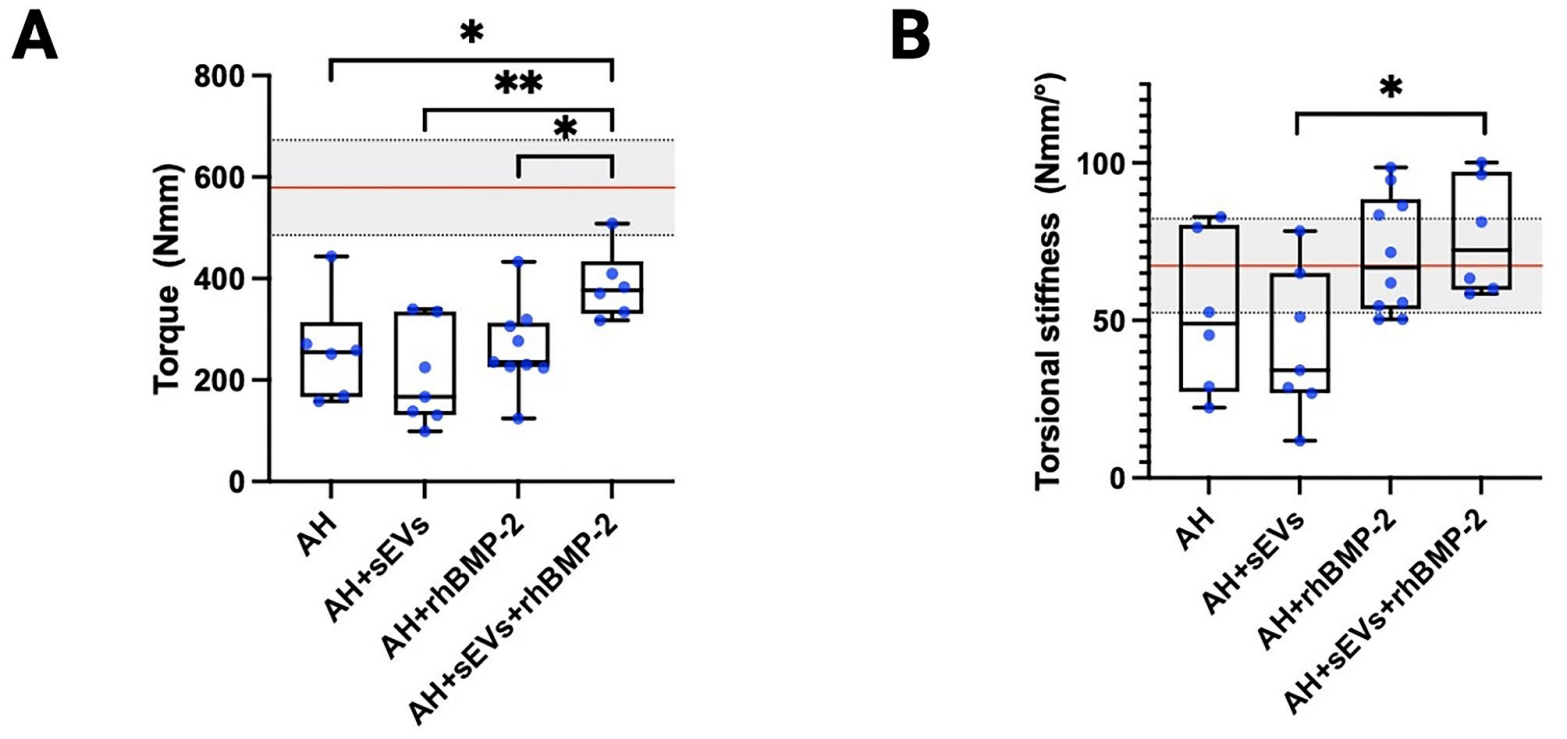
Biomechanical analysis 6 weeks post-OP. (**A**) Torque and (**B**) torsional stiffness values determined for femora treated with alginate (AH), sEVs alone, rhBMP-2 alone, or rhBMP-2 in combination with sEVs. The red line indicates mean values determined for healthy femora, with ± 1SD indicated (grey area). * = *p* < 0.05; ** = *p* < 0.01; *** = *p* < 0.005

To evaluate functional defect healing, torsional testing was performed 6 weeks after treatment, determining torque (Nmm) and the torsional stiffness (Nmm/°). Numerous samples had to be excluded from the analysis. This exclusion was based on the observation of creep, as indicated by the torque versus torsional angle function, or because the sample did not undergo fracture at the intended defect site.A significant difference in maximum torque was evident when comparing samples from the treatment groups AH and AH + rhBMP-2 + sEVs (*P = 0.0280), AH-sEVs and AH + rhBMP-2 + sEVs (**P = 0.0028), and AH + rhBMP-2 to AH + rhBMP-2 + sEVs (***P = 0.0109; Fig. 6A). Therefore, it can be concluded that the combination of rhBMP-2 and sEVs resulted in significantly more stable bony consolidation, which was closest to untreated, healthy femora. These results further substantiate the results from the histological and radiological evaluation. Surprisingly, the analysis of the torsional stiffness showed only significant difference when comparing the AH-sEVs and AH-rhBMP-2 + EVs groups (*P = 0.0152; Fig. 6B). Interestingly, all femora that had either been treated with rhBMP-2 alone or sEVs in combination with rhBMP-2 revealed stiffness values comparable to the untreated femora.

## Discussion

The objective of the current study was to explore the possible synergistic effects of human umbilical cord-derived mesenchymal stem cell-secreted extracellular vesicles in enhancing rhBMP-2-mediated bone repair in osteoporotic bone, utilizing a previously established metaphyseal rat femur defect model [17]. Based on the analysis of native X-rays, semi-quantitative and descriptive histology, micro-computed tomography (µCT), and biomechanical evaluations, we demonstrate that a single, orthotopic administration of hUC-MSC-sEVs in combination with a low dose of rhBMP-2 resulted in the most favorable outcomes for fracture healing.

Significant differences in bone healing were evident in vivo between the various treatment groups, with a superior performance of the rhBMP-2 + sEVs hydrogels. Native radiographs demonstrated mostly complete bony union, with remodeled cortices and callus formation. The same results were also observed by histological staining, where the initial fracture was almost completely resolved due to complete remodeling of the cortices via secondary fracture healing. In comparison, mean radiological and histological scores for samples in the AH, sEV, and rhBMP-2 groups showed inferior results. Most importantly, the biomechanical evaluation supported the results of the radiological and histological analyses, as the highest stability (torque) was determined for the group treated with sEVs in combination with rhBMP-2.

The distal radius, proximal humerus, proximal femur, and vertebral bodies and their trabecular or metaphyseal bone regions are especially prone to osteoporotic fractures [22, 23]. As the aging population is expected to double by 2050 and the occurrence of osteoporotic fractures is to rise in the near future, impairment of osteoporotic fracture healing is an emerging public health concern [24]. Given that the animal model employed in this investigation assesses bone repair specifically in the femur metaphysis rather than the diaphyseal femur, our study more closely mirrors the clinical scenario, as the majority of osteoporotic fractures in long bones predominantly occur in the metaphyseal region. Other animal studies have already reported upon the enhanced fracture repair and stimulation of early new bone formation with rhBMP-2 in ovariectomized rats [21, 25]. But to our knowledge this is the first study to report on the effects of combining hUC-MSC-sEVS with a low dose of rhBMP-2 to promote osteoporotic bone healing.

The improved fracture repair, particularly evident in combination with rhBMP-2 rather than with standalone sEVs, strongly implies a primary role of sEVs as facilitators in delivering rhBMP-2 more efficiently to recipient cells. In addition to their function in internal cargo transport, it is now increasingly evident that sEVs strongly bind proteins on their surface, leading to the formation of a protein corona [26–28]. Various methods have been explored for loading therapeutic cargo to sEVs, including techniques such as sonication, electroporation, freeze-thawing, and co-incubation. Notably, passive loading of sEVs through co-incubation has demonstrated efficacy in delivering anti-VEGF antibodies, effectively reducing neoangiogenesis in vivo in a rat retinopathy model [29]. In this study, passive loading via simple co-incubation at room temperature was employed, chosen for its simplicity and minimal impact on sEV membrane integrity compared to various alternative methods.

sEVs have been demonstrated to convey immune-modulatory [30], anti-inflammatory, and anti-fibrotic activities [31, 32]. Therefore, it cannot be excluded that the observed enhanced effectiveness of rhBMP-2 for osteoporotic bone repair is also a consequence of pleiotropic actions of sEVs together with an enhanced delivery of rhBMP-2. Therefore, it is also possible that the observed enhanced efficacy of rhBMP-2 in osteoporotic bone repair is a result of the pleiotropic actions of sEVs in conjunction with an improved delivery of rhBMP-2. Nonetheless, the improved effectiveness of a low dosage of rhBMP-2, observed when co-applied with sEVs in this study, is likely attributed to a more effective delivery of the osteoinductive growth factor to the target cells. EV uptake and downstream bioactive effects may be dependent on membrane-bound protein interaction with the recipient cell [33–35].

The uptake of EVs and their downstream bioactive effects have been shown to depend on membrane-bound protein interactions with recipient cells [33, 35]. Among these proteins, several tetraspanins play a crucial role, with notable examples being CD9 and CD81. These have been demonstrated to possess fusogenic properties, implicating them in processes such as phagocyte fusion. A recent study by Xu J. et al. demonstrated the essential role of CD9 or CD81 in binding and subsequent bioactivity of extracellular vesicles derived from human perivascular stem cells on recipient osteoprogenitor cells [36]. The robust expression of CD9 and CD81 in the hUC-MSC-sEVs utilized in this study indicates an effective uptake of the extracellular vesicles, and consequently, of the co-administered rhBMP-2 by osteoprogenitor cells within the bone defect.

For optimal in vivo application, it is essential to consider the pharmacokinetics and distribution of exosomes. Typically, following intravenous injection, most extracellular vesicles (EVs) have been observed to distribute to the spleen, liver, lung, and kidney within 30 min, with an approximate half-life of 3 h in the blood [37]. In our study, the local delivery of hUC-MSC-sEVs, either alone or in combination with rhBMP-2, was intended to maximize concentrations at the defect site. This approach takes into account the generally brief half-life and swift cellular uptake kinetics of sEVs. Xu CM et al. demonstrated minimal MSC-EV uptake in other organs after local intramyocardial injection of EVs in a murine model of myocardial infarction [38]. Consequently, it is highly probable that the majority of the administered sEVs remained localized at the defect site. The precise determination of biodistribution following local administration would necessitate treatments with appropriately labeled sEVs. However, it is important to note that there have been reports highlighting challenges in obtaining accurate results when employing lipophilic fluorophore staining of extracellular vesicles for uptake studies [39, 40]. Addressing this concern would be pivotal for a comprehensive understanding of the fate of locally administered sEVs and is an avenue for further investigation to ensure the reliability and accuracy of the biodistribution assessments. The current study had additional limitations. Firstly, the animals were euthanized six weeks post-femoral surgery, thereby precluding the assessment of the long-term impact of the treatments on bone remodeling. Moreover, underlying cellular and molecular mechanisms potentially driving the observed synergistic effects, beyond a potentially improved growth factor delivery, require further investigations.

The results of our study are very promising and warrant a follow-up large animal study and potentially subsequent clinical evaluations. Among the available sources of MSCs, human umbilical cord is an economically viable, productive, feasible, and universally applicable source. Numerous studies have documented the utilization of sEVs derived from hUC in the treatment of various diseases [41]. The translation of the approach outlined in this study is viable, given that rhBMP-2 (InFuse®) is clinically approved, and the small extracellular vesicles (sEVs) utilized in this study were prepared in accordance with Good Manufacturing Practice (GMP) standards. A comparable preparation has already been employed in a first-in-man study aimed at mitigating fibrotic adhesions following cochlear implant surgery [42]. . So far, the usage of rhBMP-2 applied with a collagen scaffold has been approved by the U.S. Food and Drug Administration for the promotion of spinal fusion and fracture healing [43, 44]. However, some serious adverse events have been reported after the clinical application of rhBMP-2, mainly due to the use of excessive dosages resulting in ectopic bone formation and pronounced tissue inflammation [24, 45–47]. Considering that all reported major adverse events were associated with a supra-physiological dose, our findings that the co-application of naïve hUC-sEVs allows the reduction of an effective rhBMP-2 dose [48], bears great promise to improve the safety profile of BMP-2. Importantly, owing to their natural origin, small extracellular vesicles (sEVs) exhibit high biocompatibility and limited immunogenicity. These inherent characteristics confer potential advantages over conventional synthetic drug delivery vehicles, including liposomes and nanoparticles.

## Conclusion

In summary, the simultaneous administration of rhBMP-2 and sEVs exhibited superior efficacy in healing osteoporotic defects compared to the individual administration of a low dose of rhBMP-2 or sEVs. Consequently, this approach holds the potential to reduce the dosage of rhBMP-2 required for treating osteoporotic fractures, thereby improving the safety profile associated with BMP-2 treatments.

## Electronic Supplementary Material

Below is the link to the electronic supplementary material.

Supplementary Material 1

## Data Availability

All data analyzed during this study are included in this publication. The datasets during and/or analyzed during the current study are available from the corresponding author on reasonable request.
