# Supplementary material for: Synergistic effect of umbilical cord extracellular vesicles and rhBMP-2 to enhance the regeneration of a metaphyseal femoral defect in osteoporotic rats
Source: Stem Cell Res Ther. 2024 May 20;15:144. doi: 10.1186/s13287-024-03755-8 (PMC11103988; doi:10.1186/s13287-024-03755-8)
Supplement: Supplementary file 1 — Supplementary Material 1 [file 13287_2024_3755_MOESM1_ESM.docx]

**Supplementary Material**

**µCT analysis:**

Preprocessing

Using Fiji (20), scans were rotated using TransformJ (48) so that the long axis of the bone is aligned with the Z axis of the stack with the distal side towards the high slices numbers and the posterior-anterior axis is aligned with the Y axis of the stack with the posterior side downwards. O on both the proximal and distal sides of the defect, using the polygonal and lasso selection tools, the old cortical bone was selected on 10–20 slices up to approximately 2 mm from the defect and interpolated between slices. The medullary ROI was automatically created based on the manually drawn cortical bone region by taking just the medullary border of the cortical bone and shrinking it by 8 pixels (96 µm). In slices were the medullary space ins not fully enclosed by the cortical bone region, the convex hull of the interior medullary surface was used instead and shrunk by 8 pixels (96 µm). The bone was segmented with a threshold of 500 mgHA/cm³. A mask was created where the bone is bright, and the void is dark with altered intensity values inside the drawn medullary and cortical regions.

Measurement

The mask was imported into Definiens Developer XD 2.7 (Definiens AG, Munich, Germany). A copy was created at 1/8th the resolution and classified into classes based on voxel intensity. Anything outside all ROIs was classified as void. Anything in the cortical region was classified as cortical and anything inside the medullary was classified as interior void. Using a temporary class, cortical bone was grown into void along the Z axis for 20 pixels (240 µm) and expanded in all directions by 1 pixel (96 µm). From the scene border, a temporary class was infinitely grown in X and Y directions into void. The temporary class grown from cortical bone was then assigned back to void and the temporary class from the scene border was allowed to grow further into void with a surface tension constraint of ≥ 0.49 in 5x5x5 pixel followed by ≥ 0.47 in 7x7x7. It was then expanded again by 1 pixel (96 µm) into all tissues. The resulting temporary class encompasses the entire volume outside of the cortical bone and the defect region. The exact center of the defect was measured by growing the medullary and cortical regions of the distal and proximal sides towards each other until they meet. At the largest connecting interface, the average Z position of each pixel is measured and defined as the center of the defect.

Centered on the defect, a 3.5 mm long region centered on the defect is copied to a copy 1/4th of the resolution. The resolution of the mask is increased by re-thresholding each mask and correcting the interface by region growing with an intensity constraint and surface tension ≥ 0.45 in 3x3x3. The region between the proximal and distal parts of the bone is classified as defect region. The defect center is then remeasured by computing the median of the entire volume of the defect region.

The classification is then copied back to 1/8th scale and bone and void in each region are separated into their own classes. From the stack border, a temporary class is grown with a surface tension constraint of ≥ 0.49 in 5x5x5 followed by ≥ 0.44 in 9x9x9. This temporary class represents the exterior limit of the sample and is excluded from the tissue volume for measurement. The excluded volume is synchronized to 1/4th scale.

This classification is then copied to the full resolution. In each region, bone and void is separated into their own classes. Classification errors because of the increase in resolution are corrected by region growing at the class interfaces with various surface tension and intensity constraints.

On a separate classification layer, two measurement regions are created. The central region is 1 mm along the Z axis, centered on the defect and the second region up to 1 mm adjacent to the central region.

Bone void volume were measured for medullary, defect, cortical and periosteal regions in the central 1 mm region and the adjacent 3 mm region.


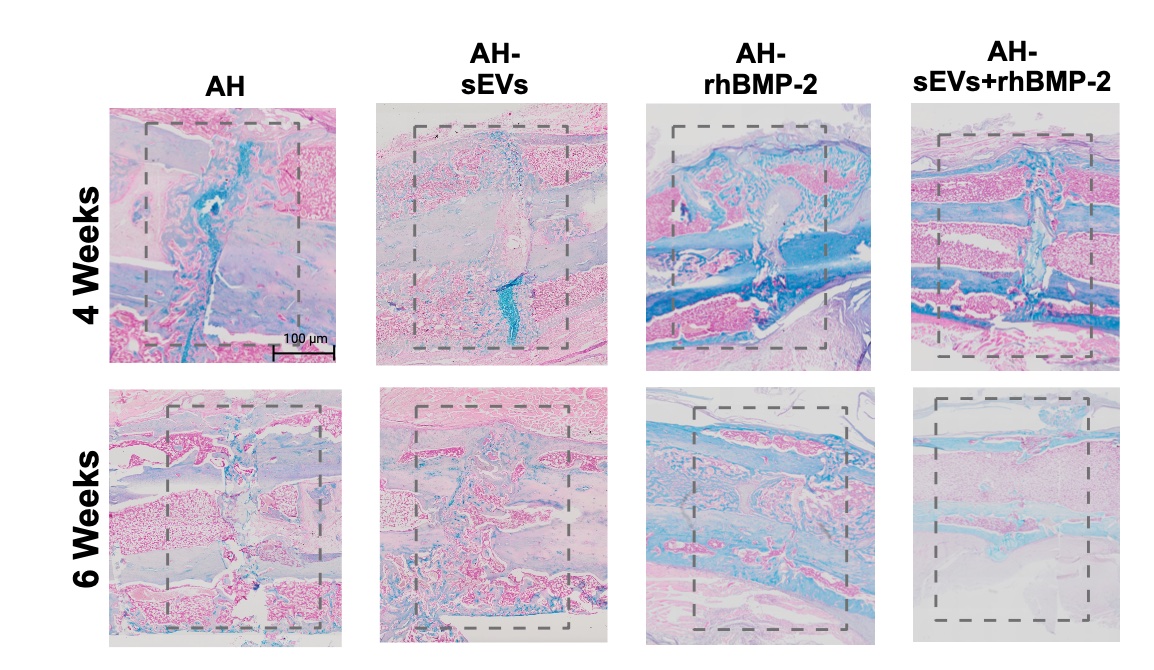

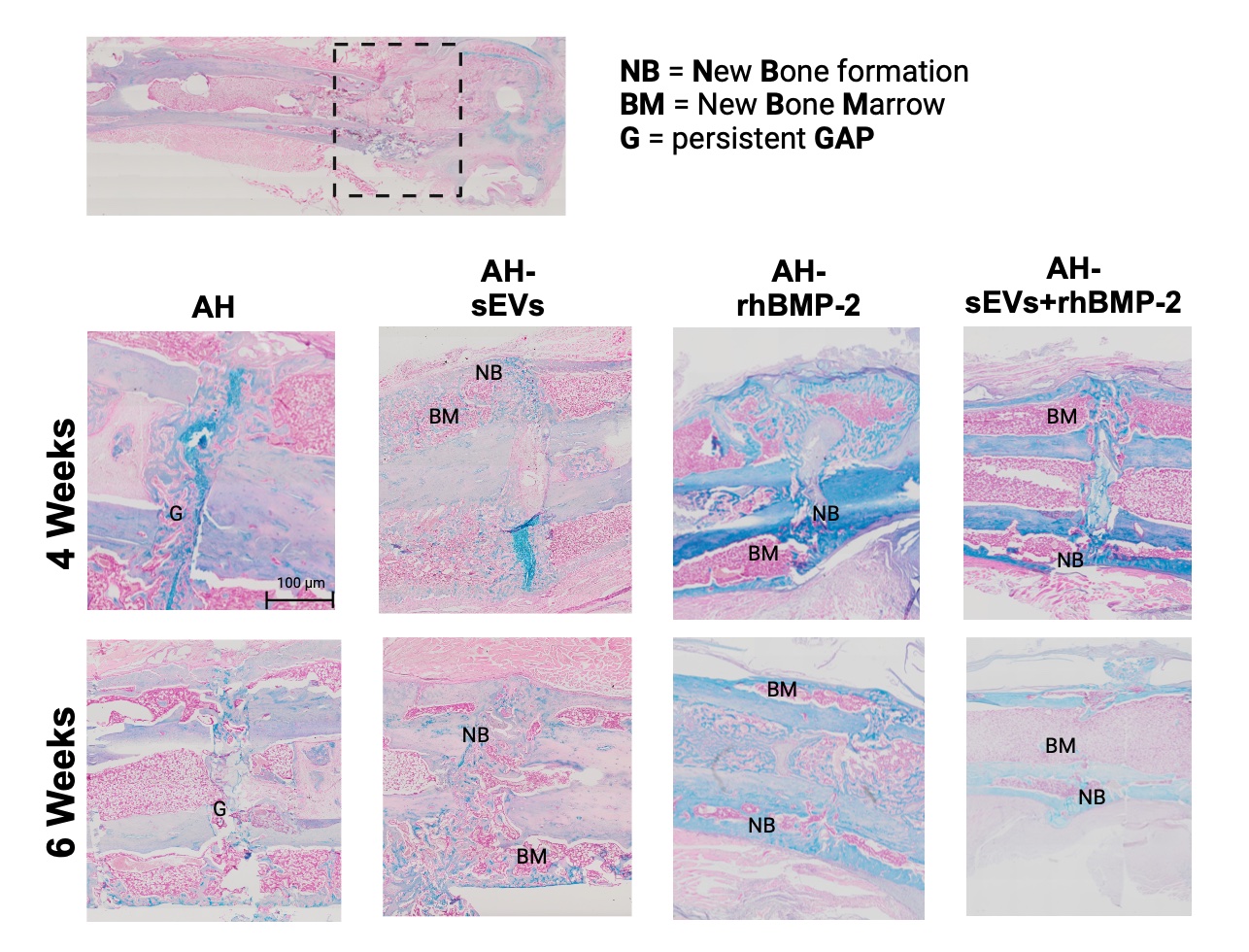


**Supplementary Figure S1**: Histological evaluation 4 and 6 weeks after treatment with Alcian blue/Nuclear fast red acid staining. Dashed lines indicate the defect area. Scale bar = 100 µm.


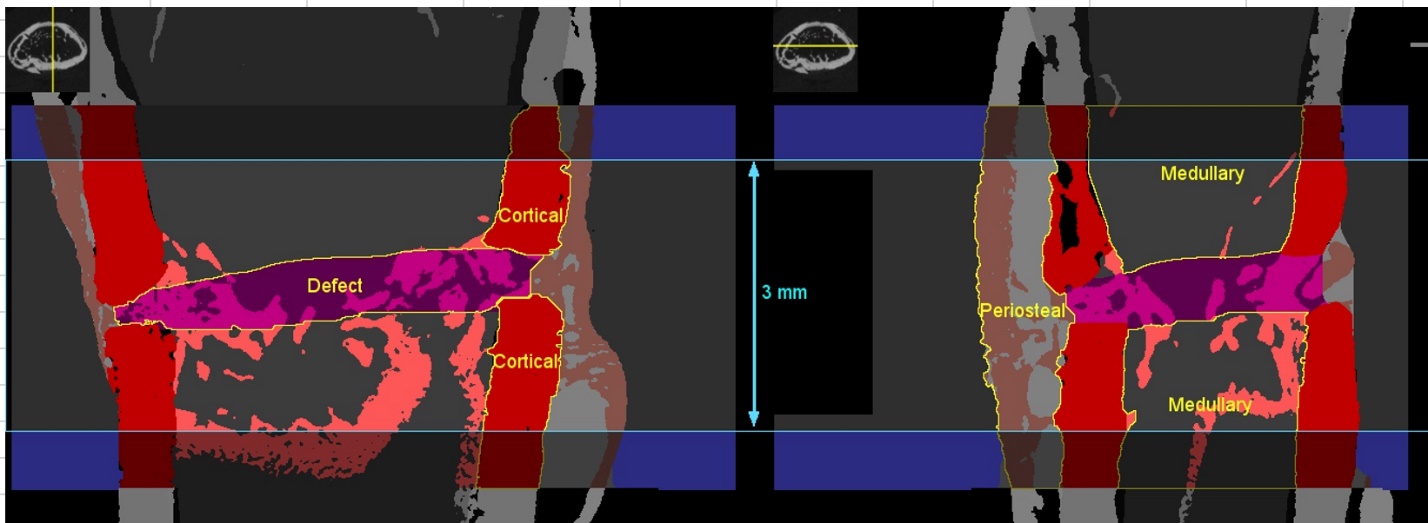


**Supplementary Figure S2**: Segmentation strategy for calculating BV/TV by µCT. New bone volume was determined by for the defect area, including callus volume and newly formed bony tissue within the medullary canal. The cortical region was excluded.
